# Inflammation-responsive biomimetic nanoparticles with epigallocatechin-3-gallate for acute lung injury therapy via autophagy enhancement

**DOI:** 10.1016/j.isci.2025.112318

**Published:** 2025-03-28

**Authors:** Ying Han, Tao Liu, Ru Wei, Chunfang Dai, Xijing Huang, Dandan Hu

**Affiliations:** 1 Guangzhou Women and Children’s Medical Center, Guangzhou Medical University, Guangzhou, Guangdong, People's Republic of China; 2 Zhujiang Hospital of Southern Medical University, Guangzhou, Guangdong, People's Republic of China

**Keywords:** Drug delivery system, Biological sciences, Biomaterials, Nanomaterials

## Abstract

Acute lung injury (ALI) is a severe inflammatory condition that can rapidly progress to acute respiratory distress syndrome, causing irreversible tissue damage. Effective anti-inflammatory and antioxidant therapies are crucial for treating ALI. We developed a dual inflammatory targeting and immune evasion capability nanoparticle, which combines epigallocatechin-3-gallate (EGCG) loaded into mercaptoketone (TK)-functionalized mesoporous silica (MSN) and coated with platelet-neutrophil hybrid membranes (PNMs). PNM-EGCG@MSN-TK nanoparticles enhance targeting to inflammatory sites and specifically remove high levels of reactive oxygen species (ROS) in cells. They also release EGCG in response to high ROS levels, improving cellular oxidative stress and enhancing autophagy in lung epithelial cells via the MAPK/BNIP3 pathway. This approach effectively ameliorates acute lung injury, suggesting a promising therapeutic strategy for ALI treatment.

## Highlights

• PNM-EGCG@MSN-TK nanoparticles enhanced targeting and ROS scavenging in ALI
• PNM-EGCG@MSN-TK reduced pro-inflammatory cytokines
• PNM-EGCG@MSN-TK activated MAPK/BNIP3 pathway to boost autophagy for ALI treatment

## Graphical abstract

**Figure:**
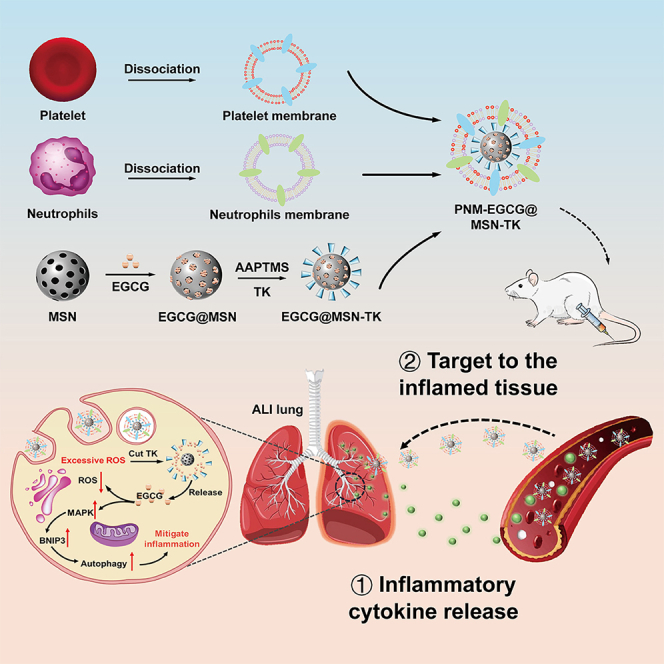

## Introduction

Acute lung injury (ALI) is a multifactorial clinical syndrome characterized by progressive inflammation-induced lung injury that mainly manifests as diffuse alveolar injury, hypoxemia, and respiratory distress.^1^ ALI has a mortality rate of 30%–40%. The pathogenesis of ALI is complex and has not been fully elucidated.^2^ Although current treatment techniques for ALI have made great progress, there is still a lack of specific therapeutic means and drugs, in addition to basic supportive treatment, and its treatment remains a challenging problem.^3^ ALI is often accompanied by strong inflammatory response and oxidative stress. It also induces mitochondrial damage and lung tissue dysfunction, resulting in the accumulation of numerous reactive oxygen species (ROS).^4^ Studies suggest that anti-inflammatory and antioxidant pathways can be modulated by endogenous or exogenous compounds, providing therapeutic approaches to control the occurrence and development of acute lung injury.

Current antioxidant and anti-inflammatory drug treatments have limited efficacy.^5^ Most of the small-molecule drugs enter cells through passive diffusion, and their distribution is not selective, which can easily lead to several side effects. Therefore, there is an urgent need to develop safe and effective drugs for ALI treatment.^6^ In recent years, advances in nanomedicine have provided greater means of removing ROS. Mesoporous silicon (MSN), with good biocompatibility and a porous structure, can adsorb drugs into the pores and is used for drug loading and controlled release. Therefore, well-designed silicon nanoparticles (NPs) have broad prospects for the treatment of ALI.^7^ However, nanomaterials are easily intercepted by macrophages, resulting in poor immune compatibility.^8^ Nature-inspired biomimetic nanocarriers have emerged as a promising approach in the field of drug delivery with the potential to enhance therapeutic efficacy. Studies have reported the use of cell membranes derived from various sources, including red blood cells, white blood cells, platelets, macrophages, and cancer cells, to fabricate these nanocarriers.^9^ These biomimetic nanomaterials can be functionalized using diverse strategies to achieve an array of beneficial properties.^10^ To surpass the limitations inherent in the use of single-cell membranes and amplify the functionality of nanomaterials, researchers have engineered specialized platforms for the preparation of hybrid membranes. These hybrid membranes serve as camouflaged NPs by integrating membranes of distinct cell types.^11^ In this study, we developed inflammation-targeting NPs with excellent targeting and immune escape capabilities by fabricating hybrid platelet-neutrophil membranes to enhance the efficacy of ALI therapy.

**Scheme 1:**
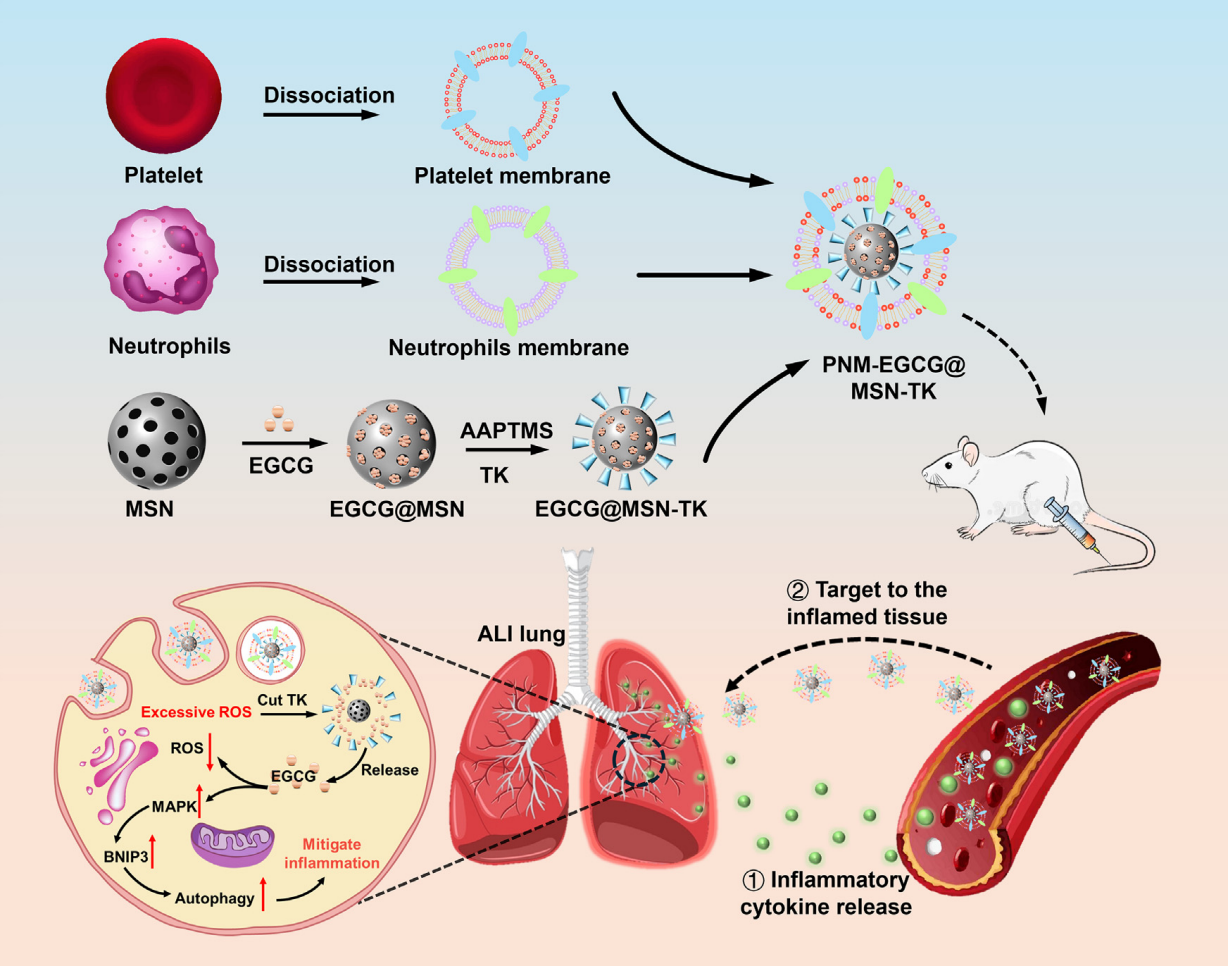
Schematic illustration of the synthesis of PNM-EGCG@MSN-TK and its application in acute lung injury (ALI) treatment

Epigallocatechin-3-gallate (EGCG) is a major polyphenol found in green tea, with powerful antioxidant activity, and has been shown to be effective in improving many diseases related to oxidative stress.^7^ EGCG can achieve antioxidant effects through the direct and interconnected removal of free radicals. In addition, EGCG increases the levels of antioxidant enzymes associated with oxidative stress, including superoxide dismutase, glutathione transferase, and heme oxygenase.^12^ However, their application is limited because of their poor stability and low bioavailability. Here, we developed a dual inflammatory targeting and immune evasion NP, PNM-EGCG@MSN-TK, in which EGCG was loaded into mercaptoketone (TK)-functionalized MSN and then coated with platelet-neutrophil hybrid membranes (PNMs). PNM-EGCG@MSN-TK NPs not only targeted the inflammatory site and cleared high levels of ROS in the cell but also upregulated the autophagy of MLE12 cells by activating the MAPK/BNIP3 pathway, thus inhibiting ALI (Scheme 1).

## Results and discussion

### Preparation and characterization of PNM-EGCG@MSN-TK

**Figure 1:**
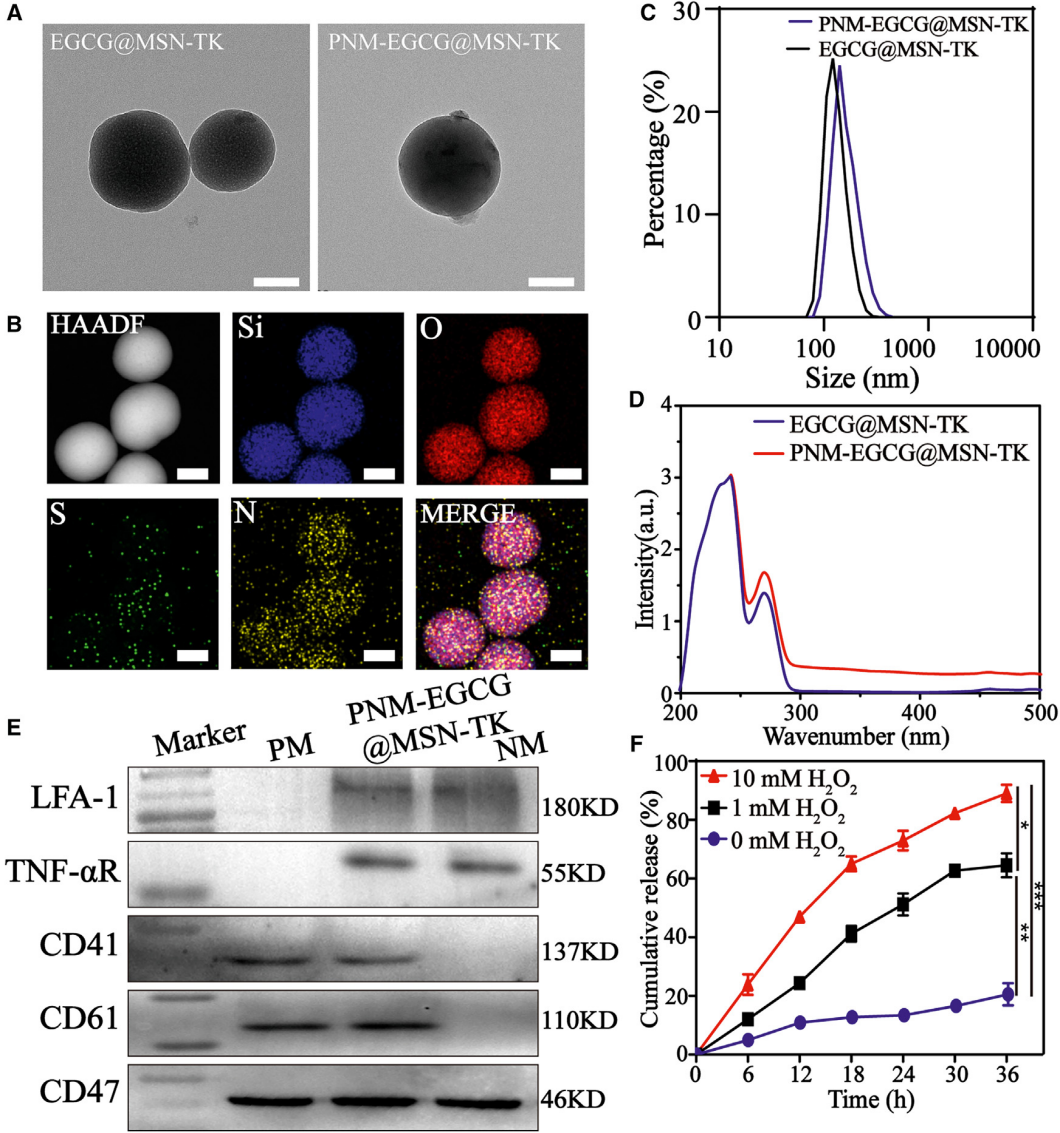
Characterization of PNM-EGCG@MSN-TK (A) PNM-EGCG@MSN-TK NPs were observed using transmission electron microscopy (TEM, scale bar: 50 μm). (B) Elemental mapping of PNM-EGCG@MSN-TK was observed by HAADF mode (scale bar: 50 μm). (C) Particle size of EGCG@MSN-TK and PNM-EGCG@MSN-TK. (D) UV-vis spectra of EGCG@MSN-TK and PNM-EGCG@MSN-TK. (E) Expression of cell membrane marker proteins in PNM-EGCG@MSN-TK using western blot analysis. (F) EGCG release from EGCG@MSN-TK. *n* = 3; data are presented as mean ± standard deviation (SD). t test was utilized for statistical analysis. ∗*p* < 0.05, ∗∗*p* < 0.01, ∗∗∗*p* < 0.001.

The morphology of the PNM-EGCG@MSN-TK NPs was analyzed using TEM (Figure 1A), and we observed that the PNM hybrid membrane was attached to the surface of EGCG@MSN-TK. The elemental mapping results showed that Si, O, N, and S were distributed in the NPs (Figure 1B), and the results showed that EGCG and mPEG-TK were successfully loaded into the NPs. DLS measurements indicated that the average diameter of PNM-EGCG@MSN-TK was approximately 122 nm (Figure 1C). As shown in Figure 1D, UV-vis spectroscopy analysis showed successful loading of EGCG into MSNs, with a load efficiency of 24.2%. Western blot analysis revealed that cell membrane protein markers characteristic of platelets and neutrophils were present on PNM-EGCG@MSN-TK, suggesting successful membrane coating (Figure 1E). Figure 1F shows the EGCG release rate with significant H_2_O_2_ dependence in the release of PNM-EGCG@MSN-TK. This was attributed to the degradation of the PNM hybrid membrane and the TK bonds on the PNM-EGCG@MSN-TK surface at high H_2_O_2_ concentrations.

### Biocompatibility of PNM-EGCG@MSN-TK

**Figure 2:**
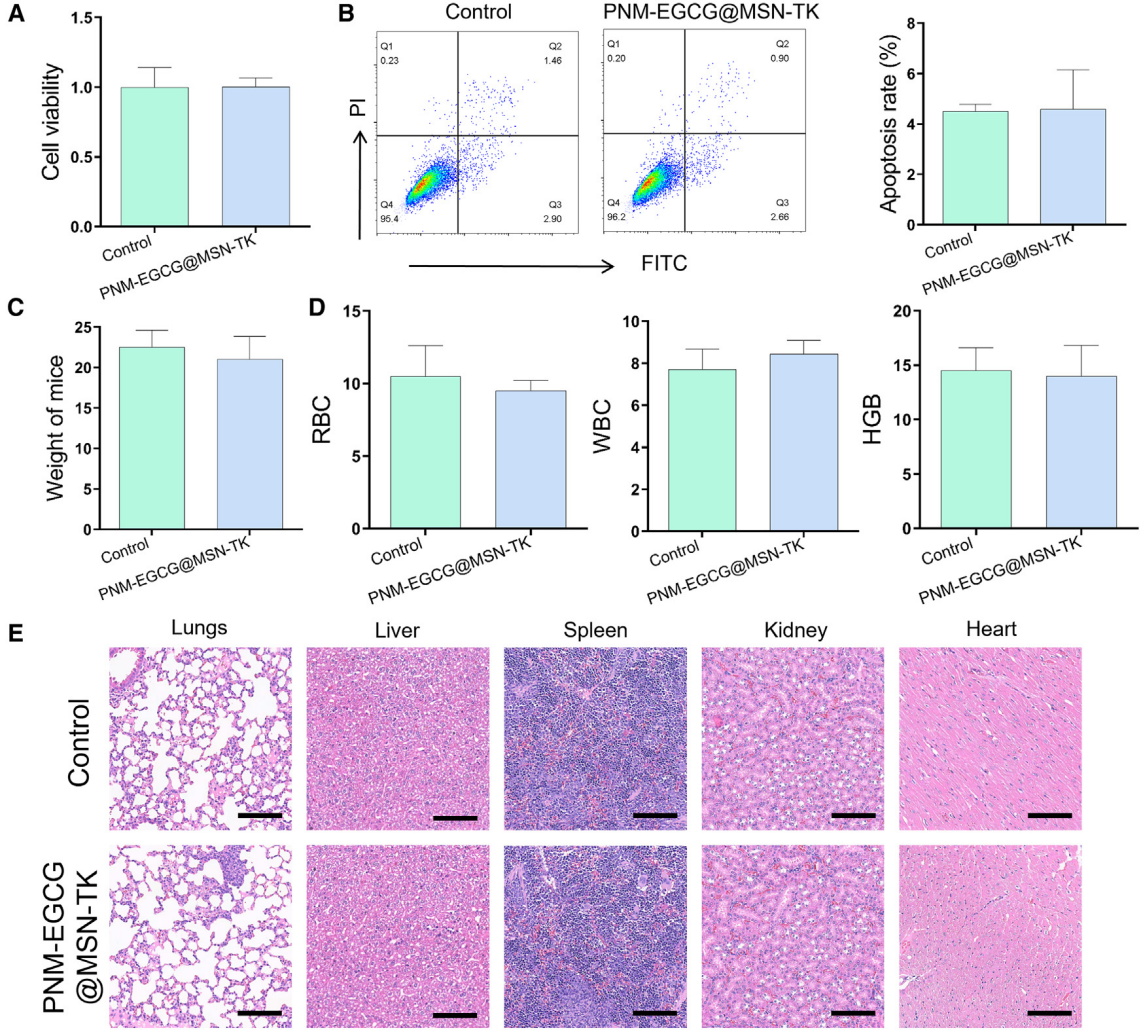
Biocompatibility of PNM-EGCG@MSN-TK (A) Viability of MLE12 cells was detected using the Cell Counting Kit-8. (B) Cell apoptosis was detected using flow cytometry. (C) Body weight of mice. (D) Routine blood analysis of RBC, WBC, and HGB. (E) Hematoxylin and eosin (H&E) staining of lung, liver, spleen, kidney, and heart (scale bar: 100 μm). *n* = 3; data are presented as mean ± SD. t test was utilized for statistical analysis.

To evaluate the biocompatibility of PNM-EGCG@MSN-TK, the cell viability of MLE12 was detected using the CCK8, and the results showed that there was no significant difference in the viability of MLE12 cells treated with PNM-EGCG@MSN-TK compared to that of normal cells (Figure 2A). In addition, the apoptosis of MLE12 cells *in vitro* was further detected by cytometry, and the results showed that the PNM-EGCG@MSN-TK group caused no obvious damage to MLE12 cells (Figure 2B). These results indicate that PNM-EGCG@MSN-TK had no obvious cytotoxicity. Furthermore, PNM-EGCG@MSN-TK was injected into the mice. After 7 days, the body weight of the mice was not significantly different between the PNM-EGCG@MSN-TK and control groups (Figure 2C). Simultaneously, the percentages of red blood cells, white blood cells, and neutrophils were analyzed using routine blood tests, and the results showed no significant difference (Figure 2D). H&E staining also showed no significant damage to the mice heart, liver, spleen, lungs, or kidneys (Figure 2E). These results indicated that PNM-EGCG@MSN-TK NPs have good biocompatibility.

### Targeting and ROS scavenging capability of PNM-EGCG@MSN-TK

Oxidative stress plays a pivotal role in ALI development. Under the stimulation of external factors, the body generates an excessive amount of ROS, leading to damage to epithelial cells and upregulation of pro-inflammatory cytokines and adhesion molecules, thereby exacerbating tissue injury.^13^^,^^14^ Moreover, the upregulation of ROS in damaged tissues can act as a chemoattractant for immune cells, leading them to migrate to the site of injury. Excess ROS enhances the ability of immune cells to invade damaged tissues.^15^ Some studies have shown that both platelets and neutrophils can rapidly migrate to sites of infection or inflammation during inflammatory responses.^16^ Based on this, an innovative strategy of using hybrid membranes formed by combining platelet and neutrophil membranes to coat NPs for drug delivery has been reported.^8^ This approach not only mimics the characteristics of natural cell membranes but also significantly enhances the biocompatibility and targeting of nanocarriers, thereby more effectively delivering drugs to inflammatory areas.

**Figure 3:**
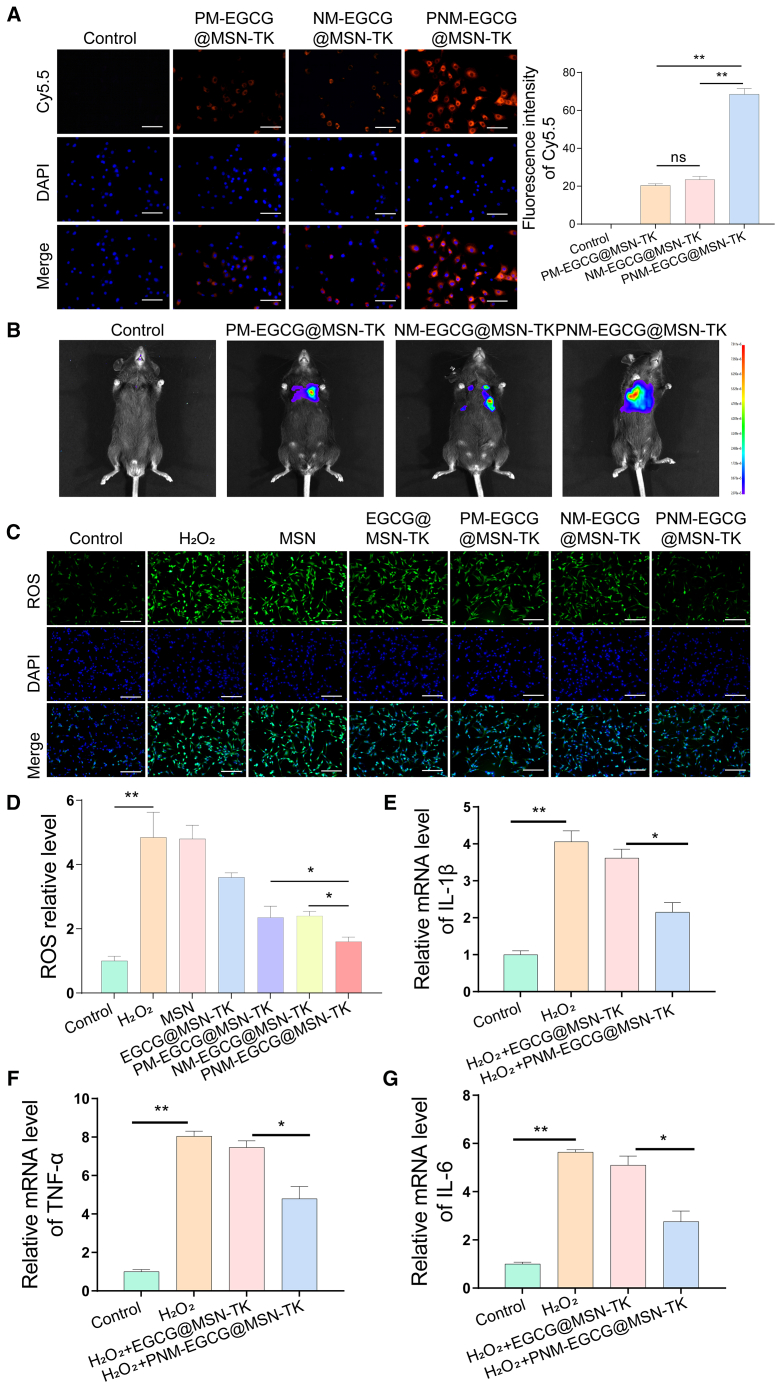
Targeting and reactive oxygen species (ROS)-scavenging capability of PNM-EGCG@MSN-TK (A) Cellular uptake of PM-EGCG@MSN-TK, NM-EGCG@MSN-TK, and PNM-EGCG@MSN-TK NPs by MLE12 cells (scale bar: 100 μm). (B) Live imaging of the ALI model mice injected with PM-EGCG@MSN-TK, NM-EGCG@MSN-TK, and PNM-EGCG@MSN-TK. (C) Immunofluorescence images of intracellular ROS after different treatment (scale bar: 100 μm). (D) Quantification of ROS levels. (E–G) Levels of IL-6,TNF-α, and IL-1β were detected using quantitative reverse-transcription (RT-qPCR) after different treatments *in vitro*. *n* = 3; data are presented as mean ± SD. t test was utilized for statistical analysis. ∗*p* < 0.05; ∗∗*p* < 0.01.

We first examined the potential effects of H_2_O_2_ in MLE12 cells. The viability of the MLE12 cells decreased following exposure to H_2_O_2_ in a dose-dependent manner. Following 24 h of exposure to 5 μM H_2_O_2_, cell viability decreased to 50%, and this concentration was utilized for further experiments (Figure S1). To evaluate the targeting of NPs *in vitro* and *in vivo*, we used equal amounts of Cy5.5/DID-labeled NPs to treat H_2_O_2_-induced MLE12 cells and LPS-induced ALI mice. As shown in Figure 3A, cells treated with PM-EGCG@MSN-TK, NM-EGCG@MSN-TK, and PNM-EGCG@MSN-TK exhibited bright fluorescence signals, and the fluorescence intensity in the PNM-EGCG@MSN-TK group was significantly higher than that in the other groups. These results indicate that the H_2_O_2_-induced MLE12 cell-targeting capability of the hybrid membrane-coated NPs is better than that of monotherapy membrane-coated NPs when exposed to cell culture medium and can be dramatically enhanced with the use of cell membrane coating. This is consistent with the results of Li et al., who found that platelet-erythrocyte hybrid-membrane-encapsulated NPs have enhanced adhesion and affinity for myofibroblasts.^17^ Moreover, *in vivo* live imaging analysis revealed that mice injected with PNM-EGCG@MSN-TK showed the brightest fluorescence signal at the lung site (Figure 3B), suggesting the good targeting ability of PNM-EGCG@MSN-TK to ALI.

To evaluate the ROS-scavenging ability of the different NPs, MLE12 cells were first treated with H_2_O_2_ and then with MSN, EGCG@MSN-TK, PM-EGCG@MSN-TK, NM-EGCG@MSN-TK, and PNM-EGCG@MSN-TK NPs. As shown in Figures 3C and 3D, DCFH (Dichlorodihydrofluorescein diacetate) was used to detect the ROS levels in MLE12 cells. The results showed that MSN could not eliminate ROS; however, EGCG@MSN-TK, PM-EGCG@MSN-TK, NM-EGCG@MSN-TK, and PNM-EGCG@MSN-TK displayed ROS-scavenging abilities. These results indicate that MSN modified with the TK bond and coated with natural cell membranes could alleviate intracellular ROS levels. In particular, the ROS-scavenging ability of PNM-EGCG@MSN-TK was significantly higher than that of the other groups. It has also been suggested that combining platelet and neutrophil membranes to coat NPs can improve oxidative stress in ALI. We also examined the effect of PNM-EGCG@MSN-TK on H_2_O_2_-induced inflammation in MLE12 cells and found that the mRNA levels of tumor necrosis factor α (TNF-α), interleukin-1β (IL-1β), and IL-6 were significantly reduced (Figures 3E–3G). These results suggest that PNM-EGCG@MSN-TK suppressed the inflammatory cytokines in MLE12 cells.

### PNM-EGCG@MSN-TK significantly enhances H_2_O_2_-induced autophagy and inhibits apoptosis in MLE12 cells

Autophagy exists widely in eukaryotic cells as a lysosome-dependent degradation pathway and plays an important role in physiological and pathological processes such as maintaining cell survival, renewal, material reuse, and stability of the internal environment.^18^^,^^19^ Moderate autophagy can promote the reuse of intracellular substances and energy, and maintain the normal function and survival of cells to a certain extent, which is an important mechanism of cell protection.^20^^,^^21^^,^^22^ EGCG is considered the most antioxidant and efficacious catechin, and previous studies have focused on its antioxidant, anti-inflammatory, and reversal of multidrug resistance in tumors.^23^^,^^24^ However, recent studies have shown that EGCG also plays a significant role in autophagy regulation. In diabetic skin injuries, EGCG promotes wound healing by enhancing autophagy in keratinocytes,^25^ whereas in vascular endothelial cells, EGCG alleviates cellular damage under oxidative stress by enhancing autophagy in vascular endothelial cells.^26^ However, the mechanisms underlying the treatment of acute lung injury remain unknown.

**Figure 4:**
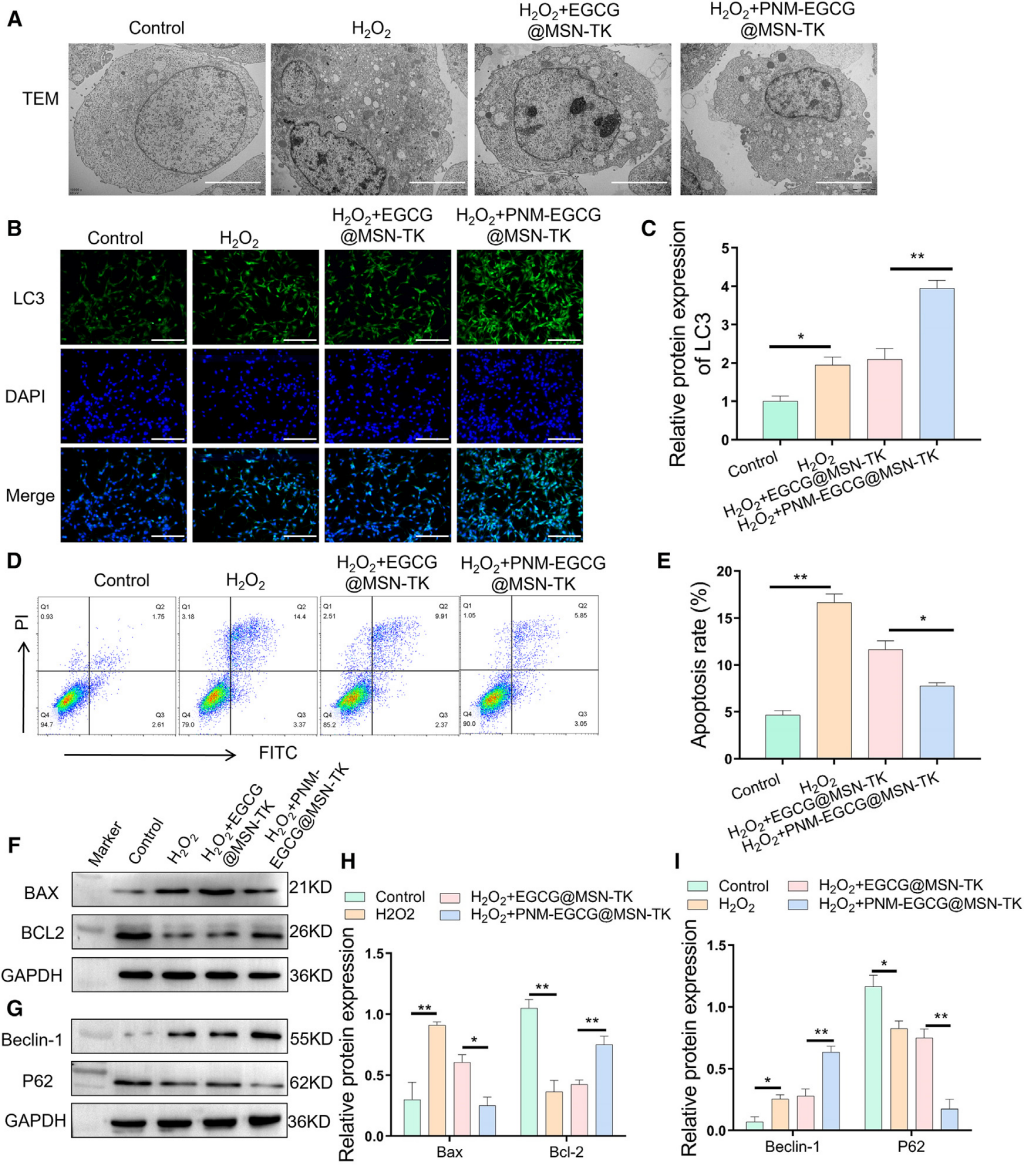
PNM-EGCG@MSN-TK enhances H_2_O_2_-induced autophagy and inhibits apoptosis in MLE12 cells (A) Autophagosomes were observed using TEM after different treatment (scale bar: 500 nm). (B and C) The expression of LC3 was detected using immunofluorescence after different treatment (scale bar: 100 μm). (D and E) Apoptosis of MLE12 cells was detected using flow cytometry after different treatment. (F and H) The expression of the apoptotic proteins BAX and Bcl2 was detected using western blotting after different treatment. (G and I) Expression of autophagy proteins Beclin-1 and P62 was detected using western blotting after different treatment. *n* = 3; data are presented as mean ± SD. t test was utilized for statistical analysis. ∗*p* < 0.05; ∗∗*p* < 0.01.

The number of MLE12 autophagosomes observed by TEM was significantly increased in the H_2_O_2_-induced MLE12 cell model treated with PNM-EGCG@MSN-TK compared with that in the EGCG@MSN-TK group (Figure 4A), suggesting that cellular autophagy was involved in the therapeutic process of PNM-EGCG@MSN-TK. Further reduction of the change in autophagy proteins LC3 and Beclin-1 similarly revealed that the PNM-EGCG@MSN-TK group was significantly higher than that in the other groups, whereas the opposite was observed for the P62 protein (Figures 4B, 4C, 4G, and 4I). These results suggest that PNM-EGCG@MSN-TK significantly enhanced autophagy in pathological MLE12 cells. To further evaluate the protective effect of PNM-EGCG@MSN-TK on H_2_O_2_-induced MLE12 cells, flow cytometry revealed that PNM-EGCG@MSN-TK significantly attenuated apoptosis (Figures 4D and 4E), whereas Bax protein levels were significantly inhibited and Bcl2 protein levels were significantly enhanced (Figures 4F and 4H). These results suggest that EGCG@MSN-TK restores cellular homeostasis and inhibits cellular damage by enhancing H_2_O_2_-induced autophagy in MLE12 cells.

**Figure 5:**
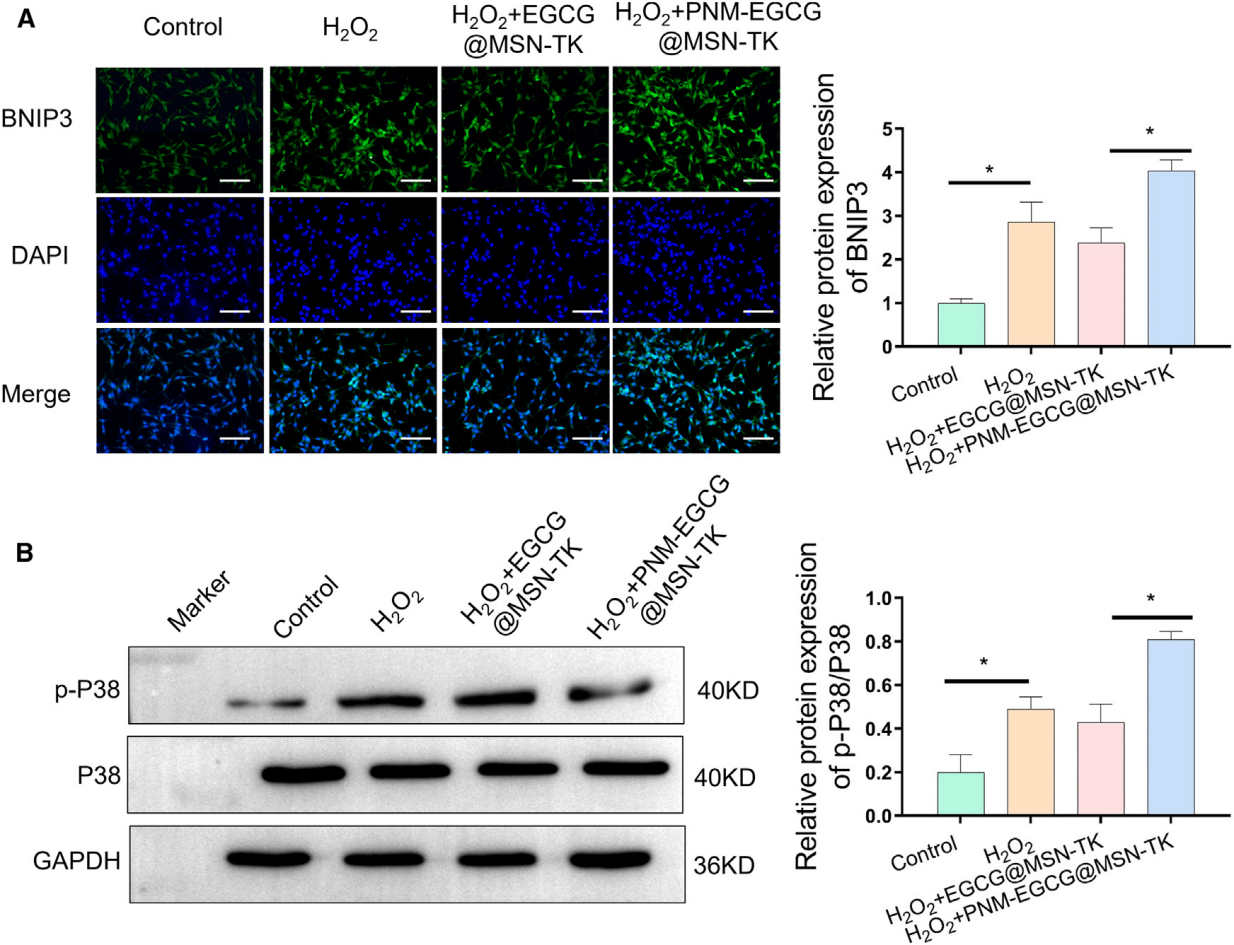
PNM-EGCG@MSN-TK regulates H_2_O_2_-induced autophagy in MLE12 cells through the MAPK/BNIP3 pathway (A) BNIP3 protein expression after different treatment methods was detected using immunofluorescence (scale bar: 100 μm). (B) The expression of P38 and p-P38 was detected using western blotting after different treatment. *n* = 3, data are presented as mean ± SD. t test was utilized for statistical analysis. ∗*p* < 0.05; ∗∗*P* 0.01.

To further explore the mechanism by which PNM-EGCG@MSN-TK enhanced cellular autophagy, we evaluated the expression of Bcl-2 19-kDa-interacting protein 3 (BNIP3). BNIP3 is a single transmembrane, hypoxia-responsive Bcl-2 family protein containing only atypical BH3 structural domains predominantly distributed on the outer mitochondrial membrane.^27^ Recently, it was reported that BNIP3 is involved in the regulation of cell survival, apoptosis, differentiation, and other biological behaviors and has been reported in a broad range of disease models, including tumor development, myocardial injury and repair, and cerebral ischemic-hypoxic damage.^28^ BNIP3 could compete with Beclin-1 for the binding site of the BH3 domain of Bcl-2, which destabilizes the binding between Bcl-2 and Beclin-1, freeing Beclin-1 and initiating autophagy.^29^ We found that PNM-EGCG@MSN-TK significantly enhanced the expression of BNIP3, suggesting that PNM-EGCG@MSN-TK promotes cellular autophagy by upregulating the expression of BINIP3 (Figure 5A). It is well known that ROS aggregation integrates intracellular signaling and regulates cellular life activities. Mitogen-activated protein kinases, including ERK, JNK, and p38, are serine-threonine protein kinases that regulate a variety of cellular activities, such as apoptosis, autophagy, and inflammatory responses.^30^ It has been noted in neuronal cells, tumor cells, and cardiomyocytes that the JNK and p38 MAPK signaling pathways regulate BNIP3 protein expression.^31^ Hence, we further examined the changes in MAPK pathway proteins and found that the expression level of p-P38 was significantly upregulated in the PNM-EGCG@MSN-TK group (Figure 5B). This indicates that PNM-EGCG@MSN-TK regulated H_2_O_2_-induced autophagy in MLE12 cells through the MAPK/BNIP3 pathway, leading to improvement of ALI.

### PNM-EGCG@MSN-TK was able to mitigate the process of ALI *in vivo*

**Figure 6:**
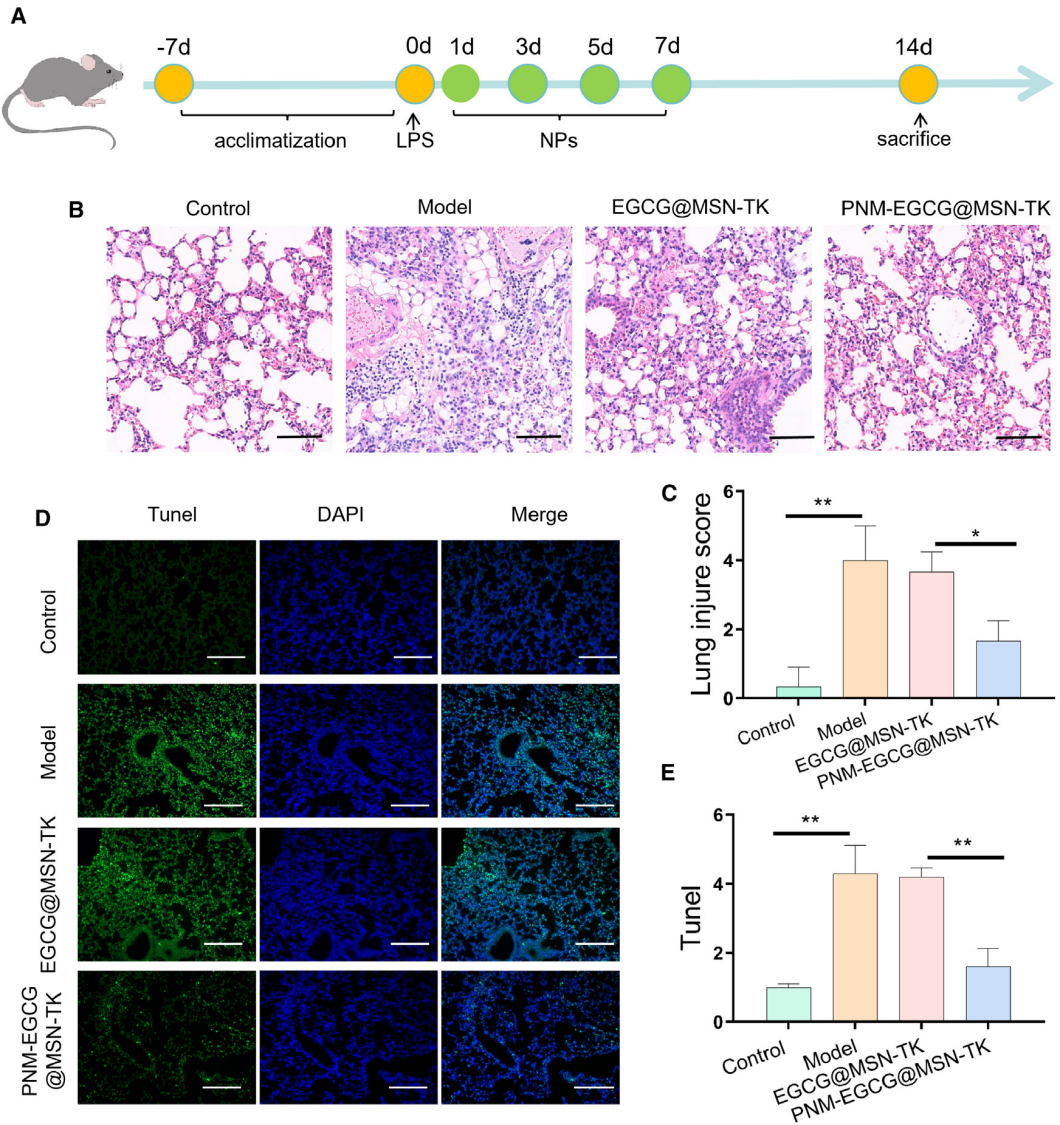
PNM-EGCG@MSN-TK mitigates ALI *in vivo* (A) Schedule of lipopolysaccharide-induced ALI model. (B and C) H&E staining was used to detect lung tissue injury after different treatments (scale bar: 100 μm). (D and E) TUNEL staining of different treatment groups (scale bar: 100 μm). *n* = 3; data are presented as mean ± SD. t test was utilized for statistical analysis. ∗*p* < 0.05; ∗∗*p* < 0.01.

To evaluate the therapeutic effect of PNM-EGCG@MSN-TK in the ALI mouse model, we first induced ALI using LPS (Figure 6A). After treatment with EGCG@MSN-TK, histopathology was used to assess tissue damage, and the effect of PNM-EGCG@MSN-TK on LPS-induced mouse lung tissue was evaluated using H&E staining. As shown in Figures 6B and 6C, the LPS group clearly showed lung interstitial thickening, loss of alveolar structure, infiltration of inflammatory cells, and hemorrhage, indicating that the ALI model was successfully constructed. Compared to the model and EGCG@MSN-TK groups, the PNM-EGCG@MSN-TK group showed significantly alleviated LPS-induced lung histopathological injury. We assessed the apoptosis of lung tissue cells using the TUNEL assay, which showed that PNM-EGCG@MSN-TK significantly inhibited the apoptosis of lung tissue cells compared with the model and EGCG@MSN-TK group (Figures 6D and 6E), indicating that PNM-EGCG@MSN-TK significantly attenuated LPS-induced lung injury in mice.

**Figure 7:**
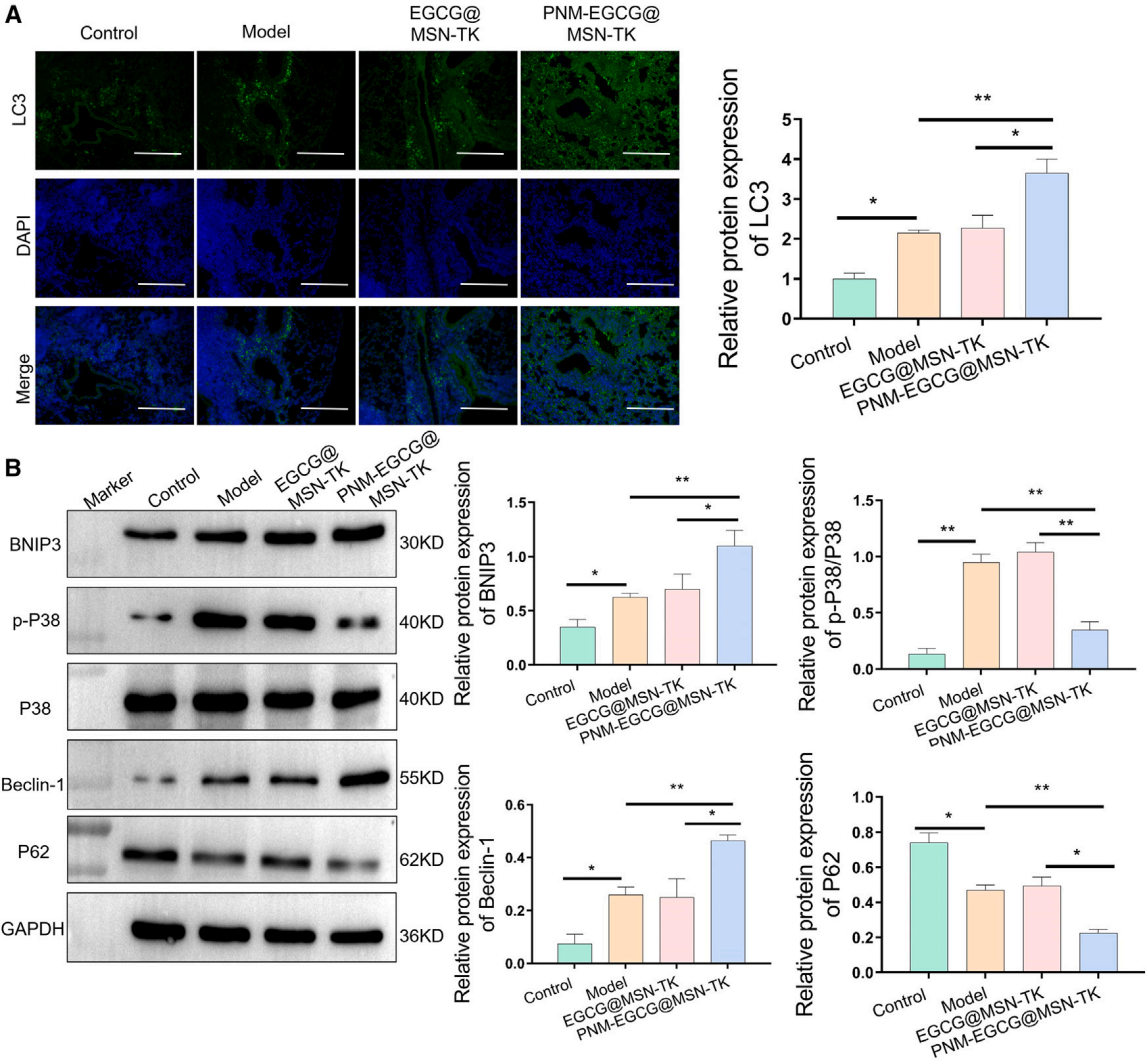
PNM-EGCG@MSN-TK enhances autophagy *in vivo* through the MAPK/BNIP3 pathway (A) LC3 expression in lung tissue was detected using immunofluorescence (scale bar: 100 μm). (B) The expression of BNIP3, p-P38, Beclin-1, and P62 in lung tissues were detected using western blotting. *n* = 3; data are presented as mean ± SD. t test was utilized for statistical analysis. ∗*p* < 0.05; ∗∗*p* < 0.01.

**Figure 8:**
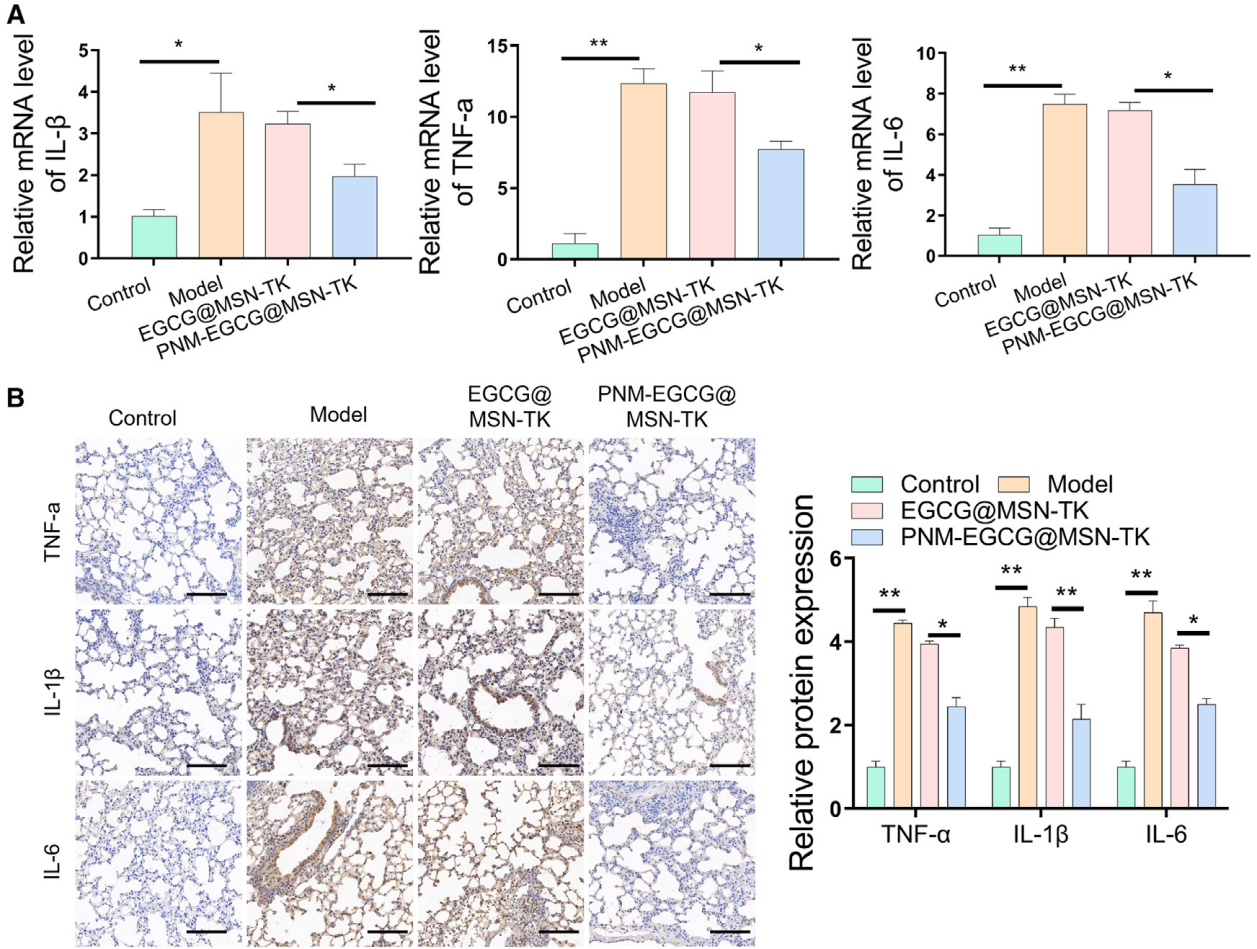
PNM-EGCG@MSN-TK improved inflammation levels in ALI mice (A) Levels of IL-6,TNF-α, and IL-1β were detected using RT-qPCR. (B) The levels of IL-6,TNF-α, and IL-1β were analyzed using immunohistochemistry (scale bar: 100 μm). *n* = 3; data are presented as mean ± SD. t test was utilized for statistical analysis. ∗*p* < 0.05; ∗∗*p* < 0.01.

Cellular autophagy plays an important role in ALI.^32^ To explore the effect of PNM-EGCG@MSN-TK on cellular autophagy *in vivo*, we detected changes in the autophagy-related markers LC3, P62, and Beclin-1 using immunofluorescence and western blotting. The results showed that PNM-EGCG@MSN-TK significantly enhanced autophagy in the lungs of LPS-induced mice (Figures 7A and 7B). To further validate the relevant mechanistic pathways of PNM-EGCG@MSN-TK in regulating cellular autophagy, we examined BNIP3 protein levels, which were significantly upregulated in the PNM-EGCG@MSN-TK group compared with those in the model and EGCG@MSN-TK groups (Figure 7B). MAPK play a key role in regulating inflammatory gene expression and cytoplasmic functional activities, which are important pathways in the eukaryotic inflammatory signaling network.^33^ To clarify the effect of PNM-EGCG@MSN-TK on the MAPK signaling pathway in an LPS-induced ALI mouse model, we used western blotting to detect changes in p-P38 in mice lung tissues. The results showed that PNM-EGCG@MSN-TK significantly enhanced the phosphorylation level of P38 (Figure 7B), indicating that PNM-EGCG@MSN-TK inhibited ALI by activating the MAPK pathway and enhancing cellular autophagy. In addition, we analyzed the levels of inflammatory factors in the lung tissue of mice, and the results showed that PNM-EGCG@MSN-TK significantly reduced the levels of TNF-α, IL-1β, and IL-6 (Figures 8A and 8B), indicating that PNM-EGCG@MSN-TK exerted a protective effect in ALI mice.

In conclusion, we successfully constructed inflammation-sensitive PNM-EGCG@MSN-TK NPs that target the inflammatory site and scavenge cellular ROS. Encapsulation of EGCG within the designed NPs enabled efficient release of EGCG in response to the inflammatory site, thereby enhancing the drug’s targeting specificity and bioavailability. In addition, PNM-EGCG@MSN-TK activated the BNIP3/MAPK pathway in MLE12 cells, which significantly increased cellular autophagy and inhibited inflammatory responses, thereby maintaining cellular homeostasis and ameliorating cellular damage. Overall, this study developed a dual inflammation-targeted drug delivery system capable of simultaneously scavenging intracellular ROS and exhibiting anti-inflammatory effects for the effective treatment of ALI, which may be a promising strategy for the treatment of ALI.

### Limitations of the study

This study primarily focuses on ROS modulation and autophagy induction; however, the precise mechanisms by which the nanoparticles interact with other molecular pathways in the inflammatory response remain unclear. Future studies should explore potential interactions with other cellular pathways to provide a more comprehensive understanding of the therapeutic effects.

## Resource availability

### Lead contact

Further information and requests for resources and reagents should be directed to and will be fulfilled by the lead contact, Dandan Hu (guohdd@126.com).

### Materials availability

This study did not generate new unique reagents or cell lines.

### Data and code availability

• The datasets generated and analyzed related to this paper are available from the lead contact on reasonable request.
• This article does not report the original code.
• Any additional information about the data reported in this paper will be shared by the lead contact upon request.

## Acknowledgments

This study was supported financially by the Science and Technology Planning Project of Guangzhou (No.202201020653) and the Project of Science and Technology Key Project for People’s Livelihood of Guangzhou, China (No.202206010060).

## Author contributions

Conceptualization, Y.H. and D.D.H; investigation, Y.H., T.L., and R.W.; data curation, Y.H., C.F.D., and X.J.H.; writing—original draft, Y.H. and D.D.H.; writing—review & editing, all authors; visualization, T.L., R.W., C.F.D., and X.J.H.; supervision, D.D.H.

## Declaration of interests

The authors declare no competing interests.

## STAR★Methods

### Key resources table

**Table:** 

REAGENT or RESOURCE	SOURCE	IDENTIFIER
**Antibodies**		

LFA-1 Antibody	ABclonal	Cat #A19009; RRID:AB_2862501
TNF-αR Antibody	ABclonal	Cat # A19127; RRID:AB_2862620
CD41 Antibody	Proteintech	Cat # 24552-1-AP; RRID:AB_2879604
CD61 Antibody	Proteintech	Cat # 18309-1-AP; RRID:AB_2128759
CD47 Antibody	Proteintech	Cat # 20305-1-AP; RRID:AB_10732838
LC3A/B Antibody	Cell signaling technology	Cat # 12741; RRID:AB_2617131
BAX Antibody	Proteintech	Cat # 50599-2-Ig; RRID:AB_2061561
BCL2 Antibody	Proteintech	Cat # 68103-1-Ig; RRID:AB_2923635
Beclin-1 Antibody	Proteintech	Cat # 11306-1-AP; RRID:AB_2259061
P62 Antibody	Proteintech	Cat # 31403-1-AP; RRID:AB_3669966
p-P38 Antibody	Proteintech	Cat # 28796-1-AP; RRID:AB_2918205
P38 Antibody	Proteintech	Cat # 14064-1-AP; RRID:AB_2878007
BNIP3 Antibody	Proteintech	Cat # 68091-1-Ig; RRID:AB_2918828
TNF-α Antibody	Proteintech	Cat # 17590-1-AP; RRID:AB_2271853
IL-1β Antibody	Proteintech	Cat # 26048-1-AP; RRID:AB_2880351
IL-6 Antibody	Proteintech	Cat # 26404-1-AP; RRID:AB_3085866
GAPDH Antibody	Proteintech	Cat # 60004-1-Ig; RRID:AB_2107436

**Chemicals, peptides, and recombinant proteins**		

Cetyltrimethylammonium Chloride	Shanghai Macklin Biochemical Co.,Ltd.	Cat # 112-02-7
Triethanolamine	Shanghai Macklin Biochemical Co.,Ltd.	Cat # 102-71-6
EGCG	Shanghai Macklin Biochemical Co.,Ltd.	Cat #989-51-5
N,N-Dimethylformamide	Shanghai Macklin Biochemical Co.,Ltd.	Cat #68-12-2
N-Hydroxysuccinimide	Shanghai Macklin Biochemical Co.,Ltd.	Cat #6066-82-6
1-ethyl-3-[3-dimethylaminopropyl]carbodiimide hydrochloride	Shanghai Macklin Biochemical Co.,Ltd.	Cat #7084-11-9

**Experimental models: Cell lines**		

MLE12	Xiamen Immocell Biotechnology Co.,Ltd	Cat #IM-M015

**Experimental models: Organisms/strains**		

C57BL/6Jmice	Guangzhou Ruge Biotechnology Co., Ltd.	NA

**Software and algorithms**		

GraphPadPrism	GraphPadSoftware,LLC	V9
Fiji ImageJsoftware	ImageJ.org	V1.53

### Experimental model and study participant details

#### Cell culture

MLE12 cells were purchased from Xiamen Immocell Biotechnology Co.,Ltd (Xiamen, China). The cells were cultured in Dulbecco’s modified eagle medium supplemented with 10% FBS, 1% penicillin, and streptomycin at 37°C and 5% CO_2_. Cells lines were tested for mycoplasma contamination before use via the Mycoplasma Detection Kit (Beyotime C0301S).

#### Animal experiment

Twenty-four male C57BL/6 mice aged 6 weeks were purchased from Guangzhou Ruige Biotechnology Co. Ltd. All mice were maintained in a specific pathogen-free facility. Food and water were provided *ad libitum* and the light/dark cycle duration was 12 h. Mice were randomly divided into four groups (n=6): control, model, model + EGCG@MSN-TK, and model + PNM-EGCG@MSN-TK. An ALI mouse model was established by administering a nasal drip of lipopolysaccharide (LPS) (20 mg/kg) at 0 day. Subsequently, NPs (10 mg/kg) were injected into the tail vein at 1, 3, 5, 7 days, respectively. On day 14, the mice were sacrificed, and mouse serum was isolated and tested using an enzyme-linked immunosorbent assay, and their lungs were weighed. The tissues samples were put into tissue fixating fluid for pathological detection, and lung injuries were scored following a previous study.^13^ Besides, some samples were stored at -80°C for RT-qPCR and western blot detection. Animal experiments were approved by the Animal Ethics Committee of Guangzhou Seyotin Biotechnology Co. Ltd (SYT2024031).

### Method details

#### Preparation and characterization of PNM hybrid membranes

Preparation of platelet membranes (PM): platelets (PLT) were isolated from whole blood using gradient centrifugation. Mouse whole blood (1 mL) was centrifuged at 100×*g* for 20 min and then at 800×*g* for 20 min. PLT were washed and centrifuged repeatedly in PBS. To obtain the PLT membranes, the PLT suspension was first frozen at -80°C, thawed at room temperature, and then centrifuged at 4000×*g* for 3 min, followed by three washes with phosphate buffered saline (PBS). PBS and protease inhibitors were mixed and added to the PLT membranes, which were then suspended in water and sonicated for 5 min. The mixture was then extruded through a mini-extruder using a polycarbonate membrane.

Preparation of neutrophil membranes (NM): The collected blood was centrifuged at 3220×*g* for 5 min to harvest cells at the interface of the 69% and 78% gradient layers, and above the 78% gradient layer. The sample was centrifuged again at 1500×*g* for 30 min, and the procedure described above was repeated to collect the cells. Roche Red Blood Cell lysis buffer was added to lyse the red blood cells. Neutrophils were washed three times with 1×PBS, resuspended, and stored at -80°C for subsequent membrane derivation.

To prepare PNM hybrid membranes, PM and NM were mixed and stirred at 37°C for 10 min. The mixture was sonicated and mechanically extruded.

#### Synthesis and characterization of NPs

Preparation of PNM-EGCG@MSN-TK: cetyltrimethyl ammonium chloride (0.2 g) and 27.5 μL triethanolamine were dissolved in 20 mL water and stirred at room temperature for 1 h. The mixture was heated to 80°C and 20 μL of tetraethyl orthosilicate was added dropwise and stirred for 1 h. After the solution cooled, the precipitate was collected by centrifugation at 10000 rpm for 10 min and washed with anhydrous ethanol to obtain non-mesoporous SiO_2_. N-MSN was obtained by dispersing 50 mg of MSN in a mixture of 200 μL deionized water, 100 μL acetic acid and 4.6 mL anhydrous ethanol, adding 2-aminoethyl aminopropyl trimethoxy silane (100 μL) for amine function, stirring for 1 h, and washing with anhydrous ethanol three times. Next, 0.1 g N-MSN was immersed in an EGCG solution (10 mL, 1 mg/mL) and stirred at room temperature for 24 h to obtain EGCG@N-MSN. Subsequently, EGCG@N-MSN (45 mg) was suspended in dimethylformamide (10 mL) and ultrasonicated for 1h, and 1-ethyl-3-[3-dimethylaminopropyl]carbodiimide hydrochloride (45 mg), N-hydroxysuccinimide (30 mg), and mPEG-TK (700 mg) were added to the suspension and stirred at room temperature for 48 h to obtain EGCG@MSN-TK. Finally, 50 μg of EGCG@MSN-TK was mixed with the prepared PNM hybrid membranes in 1 mL PBS. The mixed solution was repeatedly extruded through a mini-extruder and centrifuged at 1000×*g* to obtain PNM-EGCG@MSN-TK.

Characterization of PNM-EGCG@MSN-TK: The morphology and elements distribution of the nanoparticles were characterized using transmission electron microscopy (TEM) with a field-emission analytical electron microscope (JEM-2100F, JEOL, Japan) with HAADF-STEM mode. The samples were dispersed on a carbon film copper mesh and imaged by TEM at 200 kV to show the elemental distributions using Z-contrast. Combined with EDX technology, the X-ray spectra are analyzed to identify elements and generate elemental distribution maps. Dynamic light scattering (DLS) (Mastersizer 2000, Malvern, Worcestershire, UK) was used to measure the particle size. A Shimadzu UV-1900i spectrophotometer was used to obtain the UV-vis spectra.

The loading efficiency (LE) of EGCG was determined based on absorption in the ultraviolet (UV) range. First, the PNM-EGCG@MSN-TK dispersions were centrifuged and the supernatant was collected and diluted. The absorbance of the supernatant was measured at 275 nm using a UV spectrophotometer. LE were calculated using the following formulas:

For the *in vitro* drug release study, 2 mL of PNM-EGCG@MSN-TK suspension was placed in a dialysis bag in 10 mL of PBS with different concentrations (0, 5, and 10 mM) of hydrogen peroxide (H_2_O_2_). The release medium was collected, fresh phosphate buffered saline (PBS) was added at predetermined time intervals, and the concentration was measured using a UV-vis spectrophotometer.

#### Cell cytotoxicity assay

MLE12 cells (3×10^3^ cells, 100 μL) were seeded in 96-well plates and treated with 200 μg/mL PNM-EGCG@MSN-TK, and 100 μL of Cell Counting Kit-8 (CCK8) solution was added. The cells were incubated for 2 h, and the readings at OD_450nm_ were detected using an enzymolizer (Biotak, USA).

#### Cell uptake

MLE12 cells were seeded in 96-well plates at 1×10^4^ cells/well, treated with 5 μM H_2_O_2_ for 24 h, and treated with 100 μg/mL Cy5.5-labeled PM-EGCG@MSN-TK, NM-EGCG@MSN-TK, and PNM-EGCG@MSN-TK in PBS at 4°C. Next, the cells were stained with 4′,6-diamidino-2-phenylindole in the dark, and images were captured using a fluorescence microscope.

#### *In vivo* live imaging

DID-labeled nanoparticles (NPs) were prepared at a concentration of 100 μg/mL and injected into mice via tail vein at a volume of 100 μL. After 24 h, mice were anesthetized using 2% isoflurane and positioned in a supine orientation on a heated stage within an IVIS® Imaging System (Spectrum, PerkinElmer). Fluorescence signals of the DID-labeled NPs were detected using an excitation wavelength of 644 nm and an emission wavelength of 665 nm.

#### Detection of intercellular ROS

MLE12 cells were seeded in 24-well plates at 1×10^4^ cells/well and treated with 5 μM H_2_O_2_ for 24 h.^34^ Then, 200 μg/mL of different NPs was added for 24 h, the supernatant was discarded, and the DFCH working fluid was added. After incubation at 37°C for 30 min, cells were washed twice with PBS and observed under a fluorescence microscope (Nikon, Japan).

#### Quantitative reverse-transcription PCR (RT-qPCR) assay

Cell and tissue samples were collected and total RNA was extracted using Trizol reagent (Takara, Japan) according to the manufacturer’s protocol. Briefly, tissues were homogenized in 1 mL of Trizol per 100 mg of tissue, followed by phase separation with chloroform and RNA precipitation with isopropanol. The RNA pellet was washed with 75% ethanol, air-dried, and dissolved in nuclease-free water. For reverse transcription, 1 μg of total RNA was used as the template, and cDNA synthesis was performed using the Seyotin Reverse Transcription Kit (Seyotin, China), following the manufacturer’s instructions. The reaction was carried out at 42°C for 30 min, followed by enzyme inactivation at 85°C for 5 min. The synthesized cDNA was stored at −20°C until further use. Quantitative PCR (qPCR) was performed using the Seyotin qPCR Kit (Seyotin, China). The qPCR conditions were as follows: initial denaturation at 95°C for 5 min, followed by 40 cycles of denaturation at 94°C for 30 s, annealing at 55°C for 15 s, and elongation at 72°C for 1 h. A final dissociation curve analysis was performed to confirm the specificity of the amplification product. Relative gene expression was calculated using the 2^−ΔΔCT^ method, with GAPDH as the internal reference gene. The primer sequence was shown in Table 1.

**Table 1:** Sequence of primers used for RT-qPCR detection

Genes		Primer sequence (5′-3′)
IL-6	Forward	CAGAGGATACCACTCCCAACAG
	Reverse	CTGCAAGTGCATCATCGTTGTT
IL-1β	Forward	CTTTGAAGTTGACGGACCCCAA
	Reverse	CAATGAGTGATACTGCCTGCCT
TNF-α	Forward	CACCACGCTCTTCTGTCTACTG
	Reverse	GCTACAGGCTTGTCACTCGAAT

#### Western blot assay

Cell and tissue samples were collected and total protein was extracted from the RIPA lysate. Loading buffer was added and the mixture was boiled for 10 min, followed by SDS-PAGE and PVDF membrane transfer. The membranes were incubated with 5% bovine serum albumin (BSA) at room temperature for 1 h for blocking. Primary antibodies anti-LFA-1 (ABclonal, China), anti-TNF-α receptor (ABclonal, China), anti-CD41 (Proteintech, China), anti-CD61 (Proteintech, China), anti-CD47 (Proteintech, China), anti-Bcl2 (Proteintech, China), anti-Bax (Proteintech, China), anti-beclin1 (Proteintech, China), anti-P62 (Proteintech, China) were added and incubated at 4°C overnight. After washing three times with Tris-buffered saline containing Tween 20, membranes were incubated with the secondary antibody at room temperature and the protein signal was detected using ECL (Seyotin, China).

#### Immunofluorescence assay

The cells were seeded into 6-well plates at a density of 10^5^ cells per well for 24 h incubation. After incubation, the cells were fixed with 4% paraformaldehyde for 10 min at room temperature to preserve cellular morphology and protein localization. Following fixation, the cells were washed three times with phosphate-buffered saline (PBS, Seyotin, China). To block non-specific binding sites, the cells were incubated with 5% bovine serum albumin (BSA, Seyotin, China) in PBS at room temperature for 1 h. After blocking, the cells were incubated with Anti-LC3A/B (Cell signaling, USA), Anti-BNIP3 (Proteintech, China) specific to the target protein overnight at 4°C. The next day, the cells were washed three times with PBS to remove unbound primary antibody. Then, the corresponding secondary antibody, conjugated with Alexa Fluor 488, (Invitrogen, USA), was incubated at room temperature for 1 h in the dark to avoid photobleaching. After the secondary antibody incubation, the cells were washed three times with PBS to eliminate any unbound secondary antibody. Finally, the stained cells were examined under a fluorescence microscope (Nikon, Japan) to observe protein expression.

#### Hematoxylin-eosin (HE) staining

The lung tissue of mice was carefully harvested and immediately fixed in 4% paraformaldehyde solution for 48 h at 4°C. After fixation, the tissue was dehydrated through a graded series of alcohol solutions, starting with 70% ethanol and gradually increasing to 100% ethanol over a period of 1 h each, to remove water content. Following dehydration, the tissue was cleared using xylene, and then embedded in paraffin wax. The paraffin blocks were sectioned into 4 μm thick slices and then dried in an oven at 37°C for 1 h to adhere the sections to the slides. Afterward, the sections were dehydrated through a gradient alcohol series (100%, 95%, 80%, and 70% ethanol), each for 3 min. The tissue sections were then stained with hematoxylin for 5 min, followed by eosin staining for 2 min. The slides were then dehydrated through the gradient alcohol series again, cleared with xylene, and mounted with neutral resin. Finally, the stained tissue sections were photographed under an optical microscope.

### Quantification and statistical analysis

Statistical analysis was performed using GraphPad 9.0 for comparisons between the two groups, the t-test was used when data were normally distributed. All data are reported as mean ± SD. Three biological replicates were used for analysis unless stated otherwise, where relative statistical details of experiments can be found in the figure legend. Statistical significance was set at *P* < 0.05.
