# Supplementary material for: Inflammation-responsive biomimetic nanoparticles with epigallocatechin-3-gallate for acute lung injury therapy via autophagy enhancement
Source: iScience. 2025 Mar 28;28(5):112318. doi: 10.1016/j.isci.2025.112318 (PMC12018561; doi:10.1016/j.isci.2025.112318)
Supplement: Document S1. Figures S1 and S2 [file mmc1.pdf]

iScience, Volume *28*

Supplemental information

Inflammation-responsive biomimetic nanoparticles with epigallocatechin-3-gallate for acute lung injury therapy via autophagy enhancement

Ying Han, Tao Liu, Ru Wei, Chunfang Dai, Xijing Huang, and Dandan Hu

Supporting information

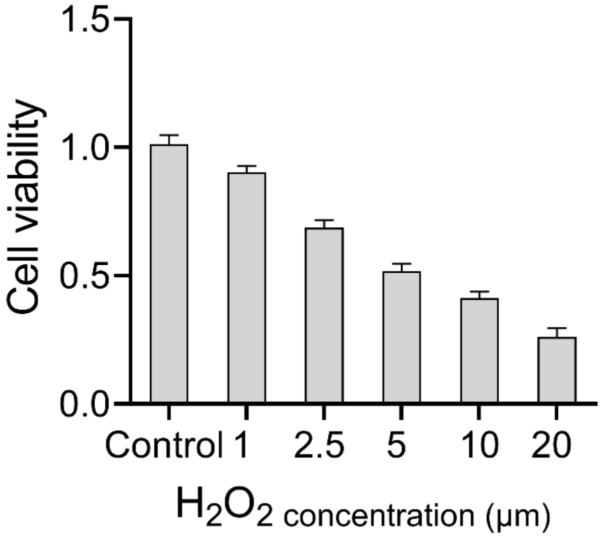

Fig.S1 Cells were exposed to various concentrations of H_2O_2 and cell viability was examined. n=3, data are presented as mean ± SD.

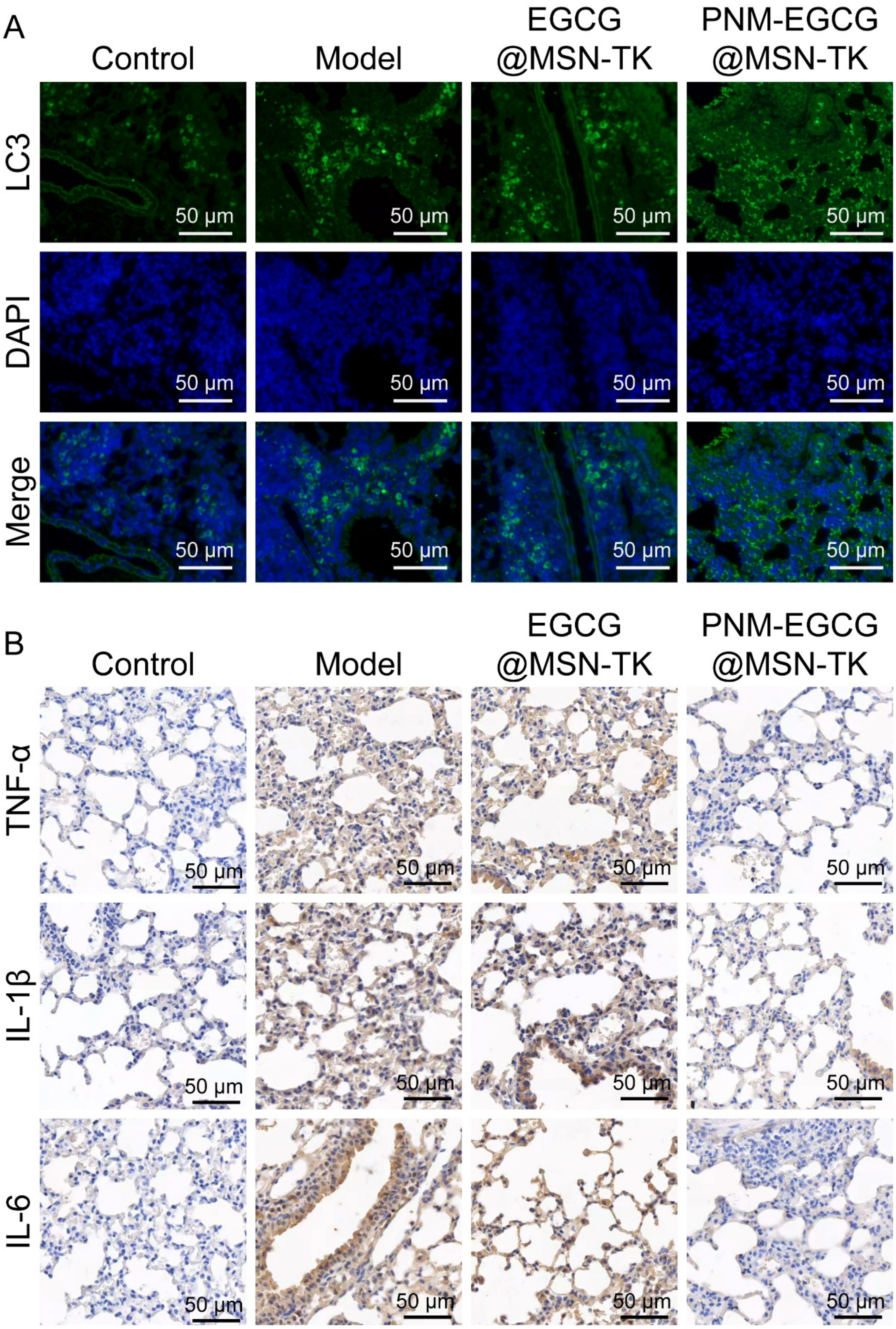

Fig.S2 Higher magnification of IF and IHC (scale bar: 50 μm).
